# Intention to get naloxone among patients prescribed opioids for chronic pain

**DOI:** 10.1186/s12954-022-00687-5

**Published:** 2022-09-22

**Authors:** Yinan Huang, Ning Lyu, Shrey Gohil, Shweta Bapat, E. James Essien, J. Douglas Thornton

**Affiliations:** 1 Department of Pharmaceutical Health Outcomes and Policy, College of Pharmacy, University of Houston, 4349 Martin Luther King Boulevard, Room 9045, Houston, USA TX 77204; 2 Prescription Drug Misuse Education and Research (PREMIER) Center, College of Pharmacy, University of Houston, Houston, TX USA

**Keywords:** Opioids, Medication safety, Naloxone, Chronic pain

## Abstract

**Background:**

Prescription opioids have been increasingly prescribed for chronic pain while the opioid-related death rates grow. Naloxone, an opioid antagonist, is increasingly recommended in these patients, yet there is limited research that investigates the intention to get naloxone. This study aimed to investigate intention toward getting naloxone in patients prescribed opioids for chronic pain and to assess the predictive utility of the theory of reasoned action (TRA) constructs in explaining intention to get naloxone.

**Methods:**

This was a cross-sectional study of a panel of U.S. adult patients prescribed opioids for chronic pain using a Qualtrics_®_^XM^ survey. These patients participated in the study during February to March 2020. The online internet survey assessed the main outcome of intention to get naloxone and constructs of TRA (attitudes and subjective norms); additional measures assessed the characteristics of patients’ opioid overdose risk factors, knowledge of naloxone, and their demographics. The relationship between TRA constructs, namely, attitudes and subjective norms, and the intention variable was examined using logistic regression analyses with the intention outcome contrasted as follows: high intention (scores ≥ 5) and non-high intention (scores < 5).

**Results:**

A total of 549 participants completed the survey. Most of them were female (53.01%), White or Caucasian (83.61%), non-Hispanic (87.57%) and had a mean age of 44.16 years (SD = 13.37). Of these, 167 (30.42%) had high intention to get naloxone. The TRA construct of subjective norm was significantly associated with increased likelihood of higher intentions to get naloxone (OR 3.04, 95% CI 2.50–3.70, *P* < 0.0001).

**Conclusions:**

Our study provides empirical support of the TRA in predicting intention to get naloxone among chronic pain patients currently taking opioids. Subjective norms significantly predicted intention to get naloxone in these patients. The interventions targeting important reference groups of these patients would have greater impact on increasing intention to get naloxone in this population. Future studies should test whether theory-based interventions focusing on strengthening subjective norms increase intention to get naloxone in this population.

## Background

More than one in five US adults were reported to have any chronic pain [1]; many Americans were prescribed opioids for treating their pain, and in 2018, over 191 million prescription opioids were issued in the US, as 15% of the population in the US were dispensed at least 1 opioid prescription, according to the Centers for Disease Control and Prevention (CDC) [2, 3]. In the meantime, opioid overdose-related mortality has quadrupled in the past 20 years [4, 5] and these overdose deaths involved pharmaceutical opioid prescriptions and/or illicit opioids [6]. In particular, prescription opioid-involved overdose resulted in over 16,000 deaths in 2020 alone, accounting for nearly 20% of all opioid overdose deaths [7]. To address the opioid crisis, the US Department of Health and Human Services emphasized the expansion and distribution of naloxone as one of five priority areas among its strategic responses [8]. Naloxone is an effective overdose reversing drug that can temporarily block the effect of opioids and can be lifesaving when an opioid overdose occurs [9]. Both federal and state organizations recommend clinicians consider co-prescribing naloxone to patients at risk of opioid overdose including people prescribed high-dose opioids for chronic pain [10–12], and “third-party prescription” laws granting naloxone accessibility to laypeople have been passed by many states [13].

An administrative data-based cohort study found that naloxone prescribing was low for patients at high risk of opioid overdose [14]. Some literature examining providers’ naloxone prescribing found that fear of offending patients [15, 17], and lack of knowledge regarding opioid overdose risk factors [16] were barriers to prescribing. In a survey of a regional sample of medical residents for assessing their naloxone prescribing practices, low proportion (15%) had prescribed naloxone despite majority (90%) showed willingness, and of note, their knowledge about the risk factors for opioid overdose is limited, a primary barrier in naloxone prescribing [16]. For example, having medical conditions of substance use disorders or depression, as well as concurrent use of benzodiazepines are common factors associated with opioid overdose, but these residents may lack the ability to link these conditions to the opioid overdosing, hampering the decision to prescribe naloxone [16]. There remains limited research on how patients perceive naloxone prescribing. In an early survey assessing drug users’ attitude to use naloxone, the majority participants held positive attitudes to administer naloxone for others during an event of overdose, potentially suggesting the benefits of take-home naloxone program [19]. In a recent survey investigating mainly chronic pain patients’ awareness involving naloxone, patients who were dispensed naloxone reported increased comfort in naloxone use and seeking opioid use counseling [18]. Another two studies found that the patients prescribed opioids for pain had positive attitudes toward being prescribed naloxone. These studies, however, have limited generalizability to the US population as they either sampled a limited number of patients from regional primary care clinics in the US [20], or enrolled participants taking opioids for chronic non-cancer pain in Australia [21]. Evidence is lacking on the willingness toward getting naloxone among patients prescribed opioids for chronic pain at a national level in the US. The limited knowledge of naloxone in patients prescribed opioid for chronic pain [22] suggests that improved understanding of patient acceptance toward getting naloxone can inform policies and practices to guide the development of interventions for increased naloxone prescribing with a goal of achieving opioid medication safety.

The aim of this study was to investigate the intention to get naloxone among a national sample of patients prescribed opioid for chronic pain and to examine whether attitudes and subjective norms guided by the theory of reasoned action model could predict the intention. Our study also described the opioid overdose risk factors and the actual experience among these patient groups of being offered naloxone prescriptions.

## Methods

### Conceptual framework

Our research utilizes the theory of reasoned action (TRA) to study the intention to get naloxone among patients on opioids for chronic pain, with chronic pain defined as pain that lasts for ≥ 3 months [28]. TRA provides a framework for attitudes, subjective norms, and behavioral intention suggesting that behavioral intention and relevant intention determine the occurrence of actual behavior, and the intention is determined by attitudes and subjective norms [23, 24]. This model is widely used in explaining behavior in public health fields [25–27].

### Study design and sample

The cross-sectional study used an online survey [29] of a national sample of U.S. adults recruited by Qualtrics (Qualtrics Inc, Provo, Utah) through multiple channels within the Qualtrics network of resources [29]. Survey invitations were sent in a general format to minimize self-selection bias. After an individual agreed to join the Qualtrics panel, a link to the survey directed them to the online questionnaire. Participants were recruited to complete an anonymous version of the survey if they were confirmed as adults aged ≥ 18 years and currently using opioids for chronic pain. Respondents were permitted to answer as many questions as they desired; however, a response was only considered complete if all questions were finished. The data was collected February–March 2020. This study was approved by the Institutional Review Board at the University of Houston.

### Survey and measures

The survey instrument was developed by adapting published surveys examining patients’ attitudes and perceptions toward naloxone [17–20, 28, 29]. The instrument was then revised based on discussion among authors to improve readability and layout. The survey was approximately 20 min in length. The final survey consisted of 44 questions, capturing patients’ risk factors for opioid overdose, knowledge of naloxone, TRA constructs (attitudes, subjective norms and intention) and demographics.

The section for opioid overdose risk factors examined patients’ condition history, chronic pain assessment, opioid pain medication use frequency, and benzodiazepine use. While there is no validated opioid overdose risk assessment tool for chronic pain patients, these potential overdose risk factors were developed based on the United States Department of Health and Human Services (HHS) guidelines regarding high-risk populations for naloxone prescribing [12]. Knowledge of naloxone was assessed based on whether respondents heard of naloxone, received counseling on naloxone, were offered naloxone, and willingness to pay for naloxone. Demographics included age, gender, race, ethnicity, education level, marital status, employment status, living area, household income and health insurance.

#### Theory of reasoned action constructs

The intention to get naloxone was assessed with nine items: “I intend/want/plan to get naloxone for myself,” “I intend/want/plan to get naloxone for my friends,” and “I intend/want/plan to get naloxone for my family members.” Participants on prescribed opioids for chronic pain may increase willingness to accept naloxone for themselves if they witness an overdose of a family/friend. Someone who may have witnessed an overdose from family and friends (third parties) can be prescribed naloxone [10], therefore, to measure broad aspects of intention, intention to get naloxone for friends/family is also measured. The mean value for three separate intention items was similar; therefore three separate “intended items” being measured were all combined and included as intention to get naloxone for any reason. Cronbach’s alpha for these items was 0.96, and the mean of the 9 items was calculated as an overall measure of intention variable.

Attitude toward getting naloxone was operationalized based on the following four items, if getting naloxone is: a good/bad thing, a harmful/beneficial practice, pleasant/unpleasant, worthless/useful. Prior literature has used these four items to measure attitude variables within the TRA model [30]. The Cronbach’s alpha for these items was 0.72 and the mean of the 4 items was calculated as an overall measure of attitude. The item 2 (if getting naloxone is a harmful/beneficial practice) and item 4 (if getting naloxone is worthless/useful) were reverse coded so that  score of higher values can  reflect a favorable attitude.

Subjective norm items assessed relevant social groups’ expectation to get naloxone; these important social groups included family, friends, physician, and pharmacists [21, 31]. Subjective norms were measured by four items: my family, my friends, my pharmacist, and my health provider who expects me to get naloxone. Higher numbers reflected higher perception of pressure toward getting naloxone from the influential groups. Cronbach’s alpha for these items was 0.96, and the mean of the 4 items was calculated as an overall measure of subjective norms.

All above TRA constructs used a 7-point Likert scale ranging from 1 representing “strongly disagree” to 7 representing “strongly agree.” The categorization of the variable scale was as follows [30]: value ≤ 3 means negative, a value ≥ 5 means positive while in the remaining value of 4 indicating moderate.

### Data analysis

A descriptive table was included for the TRA constructs. A χ2 analysis was used to analyze differences among characteristics of the respondents by high versus non-high intention group. Cronbach’s alpha was used to measure scale reliability for items for attitude, subjective norm and intention. All nine intention items were combined as an intention to get naloxone for any reason, because analysis showed that the mean for three separate intention items were similar. The Kolmogorov–Smirnov test indicating that intention was not normally distributed thus, intention was contrasted as high intention (score ≥ 5), and non-high intention (score < 5). Logistic regression analysis was used to test the association of TRA constructs and the intention variable: the measures of attitude and subjective norm were the independent variables, and intention was the dependent variable. This was performed to determine how the TRA theoretical constructs relate to the intention outcome. TRA states that attitude and subjective norm could be the lone factors predicting intention because the underlying factors influence intention indirectly through their effects on TRA constructs [32], in predicting intention in health-related behaviors [33–36]. For these reasons, we used attitudes and subjective norms to predict intention. All hypothesis tests were two sided and significance was considered at *p* < 0.05. All statistical tests were performed in SAS.

## Results

### Characteristics of participants

A total of 3440 participants clicked on the link to the survey; 944 participants started the survey, 549 of which completed the survey (15.96% response rate). The majority of respondents were aged 36–55 years (52.28%), female (53.01%), White/Caucasian (83.61%), non-Hispanic ethnicity (87.61%), married (61.20%), and employed full time (54.28%). Full information describing the sample can be found in Table 1. For *opioid overdose risk factors,* a great proportion of respondents (41.71%) had chronic obstructive pulmonary diseases, and many (43.72%) had obstructive sleep apnea. Participants (41.35%) had a non-opioid substance use disorder, 59.74% had mental health disorders, and 23.32% had cancer. The majority reported severe pain measured as pain at its worst in the last week (78.69%), at its least in the last week (51.91%), pain on the average (61.20%), or pain right now (55.19%). 42.44% used opioid pain medication near daily or daily. 54.64% of them used benzodiazepines. For *knowledge of naloxone*, less than half of the sample (48.45%) had heard of naloxone. Only 31.51% received counseling on naloxone, and likewise, 34.06% had been offered a naloxone prescription. A large majority (87.43%) showed willingness to pay for naloxone. Among those who receiving counseling on naloxone, most of them (43.35%) received counseling from more than two health providers. Most who had been offered naloxone received the naloxone prescription from the doctor who prescribed them opioid pain medication (70.05%). The majority (87.70%) filled the naloxone prescription, and 32.09% had more than two difficult reasons for filling naloxone.

**Table 1:** Characteristics of Participants with High vs With Non-high Intention to Get Naloxone (*N* = 549)

Characteristics	*n* (%)			*P* value^a^
	Total participants (*N* = 549)	Non-high intention (*n* = 382 [69.58%])	High intention (*n* = 167 [30.42%])	
***Demographics***				
**Age**				
18–35 years	153 (27.87)	92 (24.08)	61 (36.53)	< .0001
36–55 years	287 (52.28)	188 (49.21)	99 (59.28)	
> 55 years	109 (19.85)	102 (26.70)	7 (4.19)	
**Race**				
White or Caucasian	459 (83.61)	315 (82.46)	144 (86.23)	0.5478
Black or African American	55 (10.02)	41 (10.73)	14 (8.38)	
Other Racial Group^1^	35 (6.38)	26 (6.81)	9 (5.39)	
**Ethnicity**				
Hispanic	68 (12.39)	37 (9.69)	31 (18.56)	0.0037
Non-Hispanic	481 (87.61)	345 (90.31)	136 (81.44)	
**Gender**				
Male	258 (46.99)	139 (36.39)	119 (71.26)	< .0001
Female	291(53.01)	243 (63.61)	48 (28.74)	
**Education level**				
Less than high school or high school	176 (32.06)	156 (40.84)	20 (11.98)	< .0001
Undergraduate level	159 (28.96)	124 (32.46)	35 (20.96)	
Graduate level or above	214 (38.98)	102 (26.70)	112 (67.07)	
**Marital status**				
Married	336 (61.20)	204 (53.40)	132 (79.04)	< .0001
Unmarried or other status^2^	213 (38.80)	178 (46.60)	35 (20.96)	
**Employment status**				
Employed Full time	298 (54.28)	163 (42.67)	135 (80.84)	< .0001
Employed part time or other status^3^	251 (45.72)	219 (57.33)	32 (19.16)	
**Insurance**				
Employment-Based or Direct Purchase Private	254 (46.27)	168 (43.98)	86 (51.50)	0.0656
Medicare/Medicaid	257 (46.81)	182 (47.64)	75 (44.91)	
VA or CHAMPVA or uninsured	38 (6.92)	32 (8.38)	6 (3.59)	
**Household income before taxes**				
Less than $25,000	88 (16.03)	73 (19.11)	15 (8.98)	< 0.0001
$25,000—$74,999	193 (35.15)	162 (42.41)	31 (18.56)	
$75,000 to $99,999	78 (14.21)	55 (14.40)	23 (13.77)	
$100,000 to $149,999	105 (19.13)	54 (14.14)	51 (30.54)	
$150,000 or more	85 (15.48)	38 (9.95)	47 (28.14)	
**Living area**				
Urban	244 (44.44)	122 (31.94)	122 (73.05)	< 0.0001
Suburban	199 (36.25)	167 (43.72)	32 (19.16)	
Rural	106 (19.31)	93 (24.35)	13 (7.78)	
***Characteristics of Opioid Overdose Risk Factors***				
**Condition History**				
**Chronic obstructive pulmonary disease**				
Yes	229 (41.71)	103 (26.96)	126 (75.45)	< 0.0001
No	320 (58.29)	279 (73.04)	41 (24.55)	
**Obstructive sleep apnea**				
Yes	240 (43.72)	111 (29.06)	129 (77.25)	< 0.0001
No	309 (56.28)	271 (70.94)	38 (22.75)	
**Non-opioid substance use disorder**^4^				
Yes	227 (41.35)	100 (26.18)	127 (76.05)	< 0.0001
No	322 (58.65)	282 (73.82)	40 (23.95)	
**Mental health disorders**^5^				
Yes	328 (59.74)	194 (50.79)	134 (80.24)	< 0.0001
No	221 (40.26)	188 (49.21)	33 (19.76)	
**Cancer**				
Yes	128 (23.32)	52 (13.61)	76 (45.51)	< 0.0001
No	421 (76.68)	330 (86.39)	91 (54.49)	
**Pain Assessment and Opioid Pain Medication Use**				
**Chronic Pain Assessment**				
**Pain at its worst in the last week**				
Severe pain^6^	432 (78.69)	290 (75.92)	142 (85.03)	0.0164
Non-severe pain	117 (21.31)	92 (24.08)	25 (14.97)	
**Pain at its least in the last week**				
Severe pain^6^	285 (51.91)	160 (41.88)	125 (74.85)	< 0.0001
Non-severe pain	264 (48.09)	222 (58.12)	42 (25.15)	
**Pain on the average**				
Severe pain^6^	336 (61.20)	203 (53.14)	133 (79.64)	< 0.0001
Non-severe pain	213 (38.80)	179 (46.86)	34 (20.36)	
**Pain right now**				
Severe pain^6^	303 (55.19)	180 (47.12)	123 (73.65)	< 0.0001
Non-severe pain	246 (44.81)	202 (52.88)	44 (26.35)	
**Opioid pain medications frequency**				
< 1 time per month	47 (8.56)	24 (6.28)	23 (13.77)	< 0.0001
1–4 times per month	133 (24.23)	86 (22.51)	47 (28.14)	
1–2 days per week	121 (22.04)	73 (19.11)	48 (28.74)	
Near daily or daily	233 (42.44)	199 (52.09)	49 (29.34)	
**Benzodiazepines use**				
Yes	300 (54.64)	151 (39.53)	149 (89.22)	< 0.0001
No	249 (45.36)	231 (60.47)	18 (10.78)	
***Characteristics of Knowledge about Naloxone***				
**Heard about naloxone before**				
Yes	266 (48.45)	144 (37.70)	122 (73.05)	< 0.0001
No	283 (51.55)	238 (62.30)	45 (26.95)	
**Received counseling on naloxone**				
Yes	173 (31.51)	55 (14.40)	118 (70.66)	< 0.0001
No	376 (68.49)	327 (85.60)	49 (29.34)	
***Who counseled you on naloxone (n* = *173)***				
Only Physician	61 (35.26)	23 (41.82)	38 (32.20)	0.0301
Only Pharmacist	37 (21.39)	16 (29.09)	21 (17.80)	
More than 2 health providers	75 (43.35)	343 (29.09)	108 (50.00)	
**Had ever been offered naloxone**				
Yes	187 (34.06)	61 (15.97)	126 (75.45)	< 0.0001
No	362 (65.94)	321 (84.03)	41 (24.55)	
***Who offered you a naloxone prescription(n* = *187)***				
*Doctor who prescribed your opioid pain medication*	*131 (70.05)*	40 (65.57)	91 (72.22)	0.1073
*Another doctor*	*34 (18.18)*	16 (26.23)	18 (14.29)	
*Pharmacist*	*22 (11.76)*	5 (8.2)	17 (13.49)	
***Did you fill the naloxone prescription (n* = *187)***				
*Yes*	*164 (87.70)*	46 (75.41)	118 (93.65)	0.0004
*No*	*23 (12.30)*	15 (24.59)	8 (6.35)	
***What, if any, difficulties did you have filling the naloxone prescription(n* = *187)***				
*Only Pharmacy did not stock it*	*34 (18.18)*	7 (11.48)	27 (21.43)	< 0.0001
*Only Problem with insurance coverage*	*59 (31.55)*	24 (39.34)	35 (27.78)	
*Only Pharmacist didn’t know what it was*	*34 (18.18)*	22 (36.07)	12 (9.52)	
*More than 2 above reasons*	*60 (32.09)*	8 (13.11)	52 (41.27)	
**Willingness to pay for naloxone**				
Unwilling^7^	69 (12.57)	66 (17.28)	3 (1.8)	< 0.0001
Willing^7^	480 (87.43)	316 (82.74)	164 (98.2)	

The questions in italics are shown among the subgroup of patients who responded yes

*VA* veteran administration insurance, *CHAMPVA* The civilian health and medical program of the department of veterans affairs

^a^*P* values are from Pearson *χ*2 tests of association for the comparison for all baseline characteristics in terms of having high intention vs non-high intention. Significance at *P* < 0.05

^1^Includes Asian or Asian American, American Indian and Alaska Native, Native Hawaiian or Pacific Islander

^2^Includes Widowed, Divorced, Separated

^3^Includes employed part time, unemployed looking for work, unemployed not looking for work, tired, student, homemaker, self-employed, unable to work

^4^Includes Alcohol use disorder, Tobacco use disorder, Cannabis use disorder, Stimulant use disorder, Hallucinogen use disorder

^5^Includes Anxiety disorder, Mood disorder, Schizophrenia and psychotic disorder, Dementia, Eating disorder, Other mood disorder

^6^Severe pain is defined as score ≥ 7

^7^Unwilling includes strongly unwilling, unwilling, somewhat unwilling, and neither willing nor willing; Willing includes somewhat willing, willing, and strongly willing

#### Characteristics of participants by intention categories

Table 1 also presents the bivariate comparison of characteristics between participants who reported high intention and those who reported non-high intention. Overall, there were 167 individuals (30.42%) with high intention to get naloxone and 382 individuals (69.58%) with non-high intentions. There were statistically significant differences in the intention category by most demographics categories including age, ethnicity, gender, education level, marital status, employment status, household income, and living area. Significantly more participants who reported high intention were categorized as young (36.53% vs. 24.08%, *p* < 0.0001), male (71.26% vs. 36.39%, *p* < 0.0001), graduate level or above (67.07% vs. 26.70%, *p* < 0.0001), married (79.04% vs. 53.04%, *p* < 0.0001), employed full time (79.04% vs. 53.40%, *p* < 0.0001), with household income of $150,000 or more (28.14% vs. 9.95%, *p* < 0.0001), and living in urban area (73.05% vs. 31.94%, *p* < 0.0001).

Statistically significant differences in the intention variable were also observed among groups with different opioid risk factors, including medical condition history, binary category of chronic pain assessment, opioid medication use frequency and benzodiazepine use. For example, a higher percentage of the sample with COPD scored a high intention (75.45% vs. 26.95%, *p* < 0.0001) compared to those without. A higher percent of the sample with obstructive sleep apnea had a high intention (77.25% vs. 29.06% < 0.0001) compared to those without. Details about group differences in the intention category by different opioid risk factors are shown in Table 2. Furthermore, the statistically significant differences in intention groups were observed in all other naloxone knowledge-related characteristics. A higher proportion of the sample with high intention reported that they had heard about naloxone (73.05% vs. 37.75%, *p* < 0.0001) compared to those with non-high intention. Participants who had ever received counseling on naloxone were more likely to have high intention than those who did not (70.66% vs. 14.40%, *p* < 0.0001). Individuals who had ever been offered naloxone were more likely to have high intention (75.45% vs. 15.97%, *p* < 0.0001) compared to those who had not. Participants with willingness to pay for naloxone were more likely to have high intention (98.2% vs. 82.74%, *p* < 0.0001) compared to those without a willingness to pay. Details about group differences in the intention category by naloxone knowledge-related characteristics are shown in Table 2.

**Table 2:** Descriptive statistics and correlation between TRA constructs (*N* = 549)

Construct	Possible range	Mean (SD)	Attitudes	Subjective norm	Intention
Attitudes	1.0–7.0	4.74 (1.36)	1		
Subjective Norm	1.0–7.0	3.89 (1.90)	0.32	1	
Intention	1.0–7.0	3.86 (1.86)	0.3	0.83	1

#### Descriptive TRA constructs

Table 2 shows the basic statistics of three TRA constructs. The possible range was from 1.0 to 7.0. They were all positively correlated, with mild correlation between attitudes and intention, and high correlation between subjective norm and intention. Respondents had a moderate level of intention toward getting naloxone, with an average of 3.86 ± 1.86. Participants had positive attitudes toward an intention to get naloxone, with a mean value of 4.74 ± 1.36. Participants had a moderate level of influence from the relevant groups toward getting naloxone, with a mean value of 3.89 ± 1.90.

#### Logistic regression model assessing the intention to get naloxone

The odds ratio (ORs), 95% CIs, and significance levels from logistic regression on intention categories for each of the TRA constructs-attitudes and subjective norms are presented in Table 3. Subjective norm was a significant predictor of respondents’ intention to get naloxone. For each unit increase in subjective norm, the odds of having a high intention increased by approximately 3 times (OR 3.04, 95% CI 2.50–3.70, *p* < 0.0001). The attitude variable was not significantly associated with respondents’ intention to get naloxone.

**Table 3:** Logistic regression analyses of intention to get naloxone (*N* = 549)

Predictor variable	Odds ratio^a^	95% CI	*P* value^b^
Attitude	1.10	0.91–1.34	0.32
Subjective norm	3.04	2.50–3.70	< 0.0001

*CI* confidence interval

^a^Models were adjusted for theory of reason action constructs of attitudes and subjective norm

^b^Significance at *P* < 0.05

## Discussions

This is the first study quantifying the willingness to get naloxone among a national sample on opioids for chronic pain and further investigating the inherent factors of attitude and subjective norms in explaining intention based on the TRA model. This study found that subjective norm was a significant predictor of high intention to get naloxone in the chronic pain population. These study findings shield some policy implications, suggesting interventions targeting the important referents’ approval or disapproval of naloxone use would change intention that in turn affect behavior.

Overall, less than one third of the sample prescribed opioids for chronic pain reported high intention to use naloxone. This result is lower than the findings of Behar et al. that found most patients had good attitudes (57%) toward naloxone prescriptions in US regional primary care clinics [20] and is also lower than those of Nielsen et al., that showed most participants (60%) prescribed opioids for chronic non-cancer pain in Australia had positive attitudes to naloxone [21]. Our results complement the findings of these studies by looking at a broad array of US chronic pain patients prescribed opioids. This highlights the education needed regarding naloxone use in this diverse US patient population on opioids with chronic pain.

The rates of high intention were more likely among the young age group. Although focusing on illicit drug users, Nolan et al. found that a younger age was positively associated with awareness of take-home naloxone [58]. In contrast, lower rates of naloxone intention were found for groups that include female, lower education, part time or unemployed, low-income and living in rural area. This data adds to this literature by highlighting individuals who may be unaware of naloxone. It is reasonable to find that higher rates of naloxone intention were reported among participants with opioid overdose risk factors. For example, those with benzodiazepines use were more likely to indicate high intention to get naloxone than those without. Unsurprisingly, we observed that severe chronic pain was associated with the high intention variable compared to non-severe pain. It is interesting to note that a significantly higher percent of the sample with more frequent use of opioid pain medication, had a non-high intention compared to those with less frequent use of opioid pain medications; this counterintuitive finding may suggest that the sample with frequent use of opioid pain medication developed more tolerance to opioids. Additionally, we observed a gap in naloxone awareness in chronic pain patients on opioids with results indicating more than half of respondents never heard of naloxone and very few patients (about 30%) had experience with naloxone receipt/counseling. This result is inconsistent with a 2016 regional study that found a substantial proportion of patients (60%) had never heard of naloxone among 60 primary care patients on opioids [20]. Unsurprisingly, those with prior knowledge of naloxone, or with experience in receiving naloxone prescription/counseling reported high rates of naloxone intention. We think that future research should emphasize naloxone education among chronic pain population who are at high risk for opioid overdose but are unaware of naloxone.

Importantly, we found that subjective norms, but not attitudes, had a significant effect on respondents’ intention to get naloxone. This study finding adds to a body of literature indicating subjective norms were more predictive than attitudes in explaining some health-related intentions, such as use of oral contraceptive in Korean immigrant women [40], condom use in homosexually active men [33], seeking depression therapy in adolescent mothers [41], or measuring BMI from physicians [34]. It is suggested that the extent of the impact that attitudes and subjective norms had on predicting intentions were contingent upon the particular behavior under investigation while several other TRA models-based studies reported the stronger effect of attitudes on intention than subjective norms in predicting intention [42, 43]. In situations where behaviors to be examined are controversial, the influence of subjective norms in explaining intentions may be stronger than attitudes, such as use of condom/contraceptive [33, 40], or depression therapy [41]. Our study results also suggest the dominant role of subjective norm in prediction of intention outcome using collected national sample data in chronic pain population prescribed opioids may also be important in devising population-based interventions. One primary use of these TRA model-based studies is providing instrumental guidelines for effectively deploying interventions based on ascertaining the degree to which the intention is influenced by attitude, or subjective norms [27, 44–47]. Considering intention as the strong predictor of subsequent behavioral, it has been demonstrated that the interventions guided by the study findings on the premise that intention efficiently used as surrogate outcome for actual behaviors are useful in modifying behaviors [48–50].

Our results suggest that subjective norms mattered more than attitudes in influencing intention to get naloxone and this observation follows the intuition that naloxone prescription is considered acceptable among patients on opioids for chronic pain [20, 21] and barriers to get naloxone primarily include perceived social pressure in requesting naloxone, and consequences of asking for naloxone. These findings offer additional insights from application viewpoints. First, it suggested that methods targeted toward alleviating social pressures could have a greater impact on increasing the use of naloxone compared to strategies that are solely aimed to change patients’ attitudes. Second, combining this study finding with evidence on the roles of social groups in patient’s acceptance to naloxone could drive theory-based interventions to improve intentions to get naloxone in patients with chronic pain prescribed opioids. Providers could be a strong influential group in enhancing patients’ intention to get naloxone amid the naloxone co-prescribing policy [12]. The important role of health care professionals in supporting education about naloxone services with non-judgmental approaches to promote opioid medication safety and relieve patient fear [51–53], and given the lack of readiness in pharmacist to prescribe naloxone [57], interventions targeting pharmacist to improve their awareness and willingness to prescribe naloxone is also needed for patient’s access to naloxone.

Family and friends are strong referent groups in this scenario, as the implementation of “third party prescription” legislation allows anyone with loved ones at risk of opioid overdose to access naloxone [54]. Indeed, some studies show that many programs about opioid overdose education and naloxone distribution training have begun to engage family or friends of opioid users [55, 56]. The evidence and our findings suggest that encouraging family and friends to accompany their loved ones to participate in education programs about naloxone distribution and opioid medication safety could be useful in increasing intention to get naloxone and preventing opioid medication related risk in this patient population.

A few limitations exist in this study. First, this is a cross-sectional study, hence causality between subjective norms and attitudes with intention could not be determined. Second, all responses were self-reported, and it may cause social desirability bias, as respondents usually tend to answer in a more socially acceptable manner. Third, underlying factors including patients’ demographics, medication/condition history or experience with naloxone prescription/counseling were not included in the analysis examining the association between attitudes and subjective norms on intention; as per Ajzen, if these background factors are considered to have an indirect impact on intention outcome, these factors may not need to be accounted for in the model. Nevertheless, we incorporated a bivariate analysis to examine the subgroup differences in individuals with high intention compared to those without. Fourth, although the Qualtrics report that the panels surveyed were representative of national U.S. samples, the external validity may be limited due to the nature of a commercial panel recruited by Qualtrics. For example, the recruitment of participants mainly targeted participants with access to the Internet; thus, our study findings may not be generalizable to those without Internet access.

Despite these limitations, this study provides preliminary evidence on the impact of social norms on predicting intention in patients prescribed opioid for chronic pain toward obtaining naloxone. Our study participants’ characteristics were similar to those reported by studies involving a national sample with chronic pain [37–39], and this helped to strengthen the external generalizability of our study findings. Our results emphasized the role of subjective norms in predicting intention to get naloxone in patients prescribed opioids. Providers and pharmacists can be influential social groups in enhancing patient knowledge of naloxone, and framing an unbiased discussion on the utility of naloxone. As a part of social groups, family and friends can and should influence patients’ intention to get naloxone by accompanying these patients to educational programs about overdose risks and use of naloxone. This finding is particularly useful in guiding intervention programs to promote naloxone use with a goal of opioid medication safety in this population.

## Conclusions

The TRA was used as a theoretical framework to study factors influencing patients’ intention to get naloxone and the role of direct measures of attitude and subjective norms in predicting intention. Subjective norms significantly predicted intention to get naloxone in chronic pain patients on prescription opioids. Considering today’s concern for prescription opioid overdoses, the distribution of naloxone is a suggested intervention in the patient prescribed opioid for chronic pain. Future studies should test whether increasing subjective norms increase the likelihood of actual behavior of getting naloxone in this population.

## Data Availability

The corresponding author can provide the material used and data analyzed on request.

## Abbreviations

TRA: Theory of reasoned action
HHS: Health and human services
OR: Odds ratio
VA: Veteran insurance
CHAMPVA: Civilian Health and Medical Program of the Department of Veterans Affairs
